# Bias-corrected-based collaborative filtering recommendation (Bias-Corr-CF)

**DOI:** 10.1371/journal.pone.0324173

**Published:** 2025-06-30

**Authors:** Tu Cam Thi Tran, Hiep Xuan Huynh

**Affiliations:** 1 Can Tho University (CTU), Can Tho, Vietnam; 2 Vinh Long University of Technology Education (VLUTE), Vinh Long, Vietnam; 3 CTU Leading Research Team on Automation, Artificial Intelligence, Information Technology and Digital Transformation (CTU-AIMED), Can Tho, Vietnam; University of South Australia, AUSTRALIA

## Abstract

The goal of the collaborative filtering problem is to find accurate and efficient mappings from previously rated data at items of the users. Improving item-based collaborative filtering (IBCF) and user-based collaborative filtering (UBCF) involves understanding the mathematics of distance measures and finding the right balance between calculating similarity and providing recommendations very accurate. However, the popular distance measures for recommendation models only focus on measuring pairwise rating values between one user and another, or between one item and another. In this article, the authors have proposed a new recommendation model, which consists of building a collaborative filtering model with the bias-corrected distance correlation statistic. The correlation method focuses on measuring the rating values of one object with all ratings of the other object; the Bias-Corrected Distance Correlation (BCDCOR) provides an improved estimate of the distance correlation; it corrects the bias present in the original distance correlation. Experimental results are developed on the Jester5k dataset, with two popular evaluation methods for the recommendation models, namely precision and recall values. The experimental results show that with the Bias-corrected-based recommendation model between users and users, the Precision and Recall values of the proposed model are higher than those of the compared collaborative filtering recommendation systems.

## Introduction

The previous collaborative filtering models [1,2] have made many efforts to build the effective mechanisms to recommend the items (such as: the movies, the products, the songs, the videos, the news, etc.) to the users based on other users’ preferences, Neural graph based recommendation models [3,4], self-supervised learning based collaborative filtering [5–7] the ultimate goal is to increase the user’s satisfaction and corresponding revenue for the providers. Because they have to calculate the similarity between objects (between the users, or between the items), the collaborative filtering recommendation systems [8,9] often use the measures such as: Pearson, Cosine, Euclidean, Jaccard, etc. [2,10]. However, the measures are only based on the general principle that they use the rating values of two objects in pairs [11], and it only care about linear issues, but it does not care about nonlinear issues, with the distance are calculated distributed from a rating value of this object to all rating values of other object. In addition, the goal of the recommendation models is to find solutions to improve the model’s performance, improve its accuracy, and provide the appropriate suggestions.

In this paper, we proposed the new recommendation models to use user-based and item-based collaborative filtering models [1,9] as a general basis [10,11]. More specifically, to develop the collaborative filtering model, we combined collaborative filtering with bias-corrected distance correlation Statistic [12,13]. To learn user preferences, power distance correlation measures [14,15] the compatibility between users and between items, then the compatibility matrix, using the results generated to find the missing rating.

The using of collaborative filtering in general [11] and Bias-Corrected Distance Correlation statistics [16] in particular provide a promising framework for the recommendation task with the following two reasons: Firstly, collaborative filtering predicts preferences and user needs without understanding the product. Recommendations based on similar user experiences can suggest the appropriate new products. Collaborative filtering [10] is suitable for large systems with many user reviews. Exploiting the capabilities of the model to train a general collaborative filtering model [8,9] with both high accuracy and high scalability. Secondly, recent advances in energy distance have enabled rapid computation of the correlations between the objects, making it feasible even for the datasets bias. The correlations distance measures the degree of association between two random variables, considering both linear and nonlinear relationships. Unlike traditional correlation (such as Pearson correlation), which only captures linear dependence. Therefore, a bias-corrected distance correlation Statistic is proposed, which is expected to perform well, and has been trained from the recommendations of the research team. Contributions of the paper:

- First, the bias-corrected distance correlation Statistic method is proposed to measure the compatibility between two users and two items. This method helps to express the variation relationship between the energy measures.

- Second, the collaborative filtering recommendation model is proposed based on the correlation between the bias-corrected distance and the index in the recommendation model for both users and items. This model solves the recommendation problem when the conditional and decision attributes are on the same object.

- Third, the energy package has been successfully integrated into the recommenderlab package in R language. In addition, tools have been built including the functions: data processing, calculating the compatibility of two objects based on energy statistics, functions for building and evaluating collaborative filtering recommendation models using the energy method.

The structure of this article is organized as follows. Section “Related works" provides a brief introduction to user-based collaborative filtering (UBCF), item-based collaborative filtering (IBCF), and it presents the bias-corrected distance correlation statistical method. In Section “Modeling", we built the model and the recommendation system algorithm with the bias-corrected correlation distance approach. Section “Experiment" is devoted to clarifying the proposed model with two scenarios; these two scenarios are built in the experimental results section of the article. In Section “Discussion" and “Conclusion", we discuss the ideas presented and conclude.

## Related works

### Collaborative filtering recommendation

**User-based collaborative filtering (UBCF)** [1,9] is a technique used to predict items that a user might like based on the ratings given to those items by other users who have similar tastes. The steps involved in this approach:

1. Finding Similar Users [17,18]: Calculate the compatibility between the target user (called *U*_*a*_) and other users *U*_*i*_. Compatibility can be determined using the bias-corrected distance correlation statistic [19,20], based on the ratings given by users to common items. The more compatible a user is to the target user, the higher their weight-age in the recommendation process.
2. Predicting Missing Ratings [2,10]:Once compatible users have been identified, the missing rating can be predicted for an item that the target user *U*_*a*_ has not rated.

**Fig 1:**
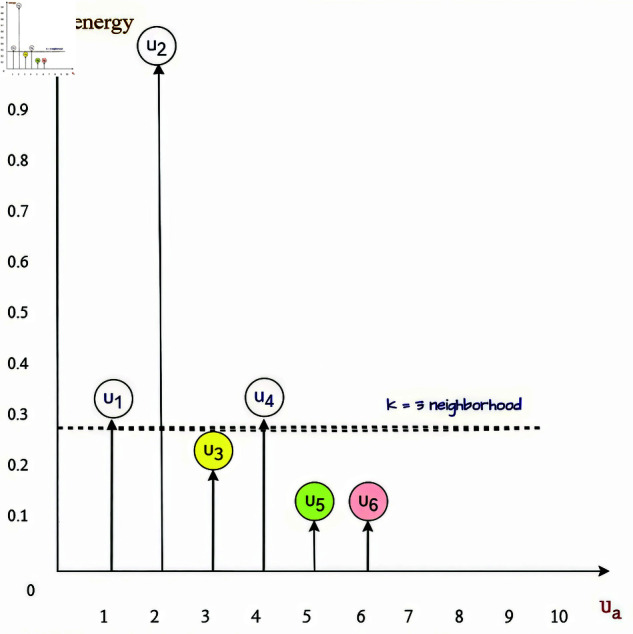
Bias-corrected distance correlation statistic with CF.

Fig 1, general description when k-nearest neighbors *knn = 3*, three users with the lowest energy are selected from user *U*_*a*_ to other *U*_*i*_ users. *Knn* is understood as an algorithm to find the k nearest data points (neighbors) in the training set. Then, determine the class of the new point based on the majority of those neighboring points.

**Item-based collaborative filtering (IBCF)** [1,2] is a recommendation technique that plays a crucial role in personalized recommendations. IBCF, also known as item-item collaborative filtering, was developed by Amazon in 1998. It’s a model-based algorithm for making recommendations. Instead of matching users to similar customers, IBCF focuses on finding similar items based on the items users have already liked or interacted with positively. The core idea is to suggest an item to a user based on the items they have previously consumed.

For example:

Suppose that a user Jone has liked or watched movies A, B, and C.

IBCF would then search for other movies similar to A, B, and C.

If it finds that movie D is very similar to movie C, it would recommend movie D to Jone due to for similarity.

The process involves calculating compatibility between different items in the dataset using various compatibility measures. These compatibility values are then used to predict the ratings for pairs of user-item that are not present in the dataset. The IBCF is to identify items that are similar to the ones a user has already enjoyed.

Improvement of IBCF and UBCF involves understanding the mathematics behind it and finding the right balance between compatibility computation and recommendation generation.

### The bias-corrected distance correlation statistic

Distance correlation [12,13] is a statistical measure that quantifies the association between two random variables in a multivariate setting. It captures both linear and nonlinear dependencies, making it a versatile tool for exploring relationships in data. However, like any statistical estimator, the standard distance correlation can be biased, especially when sample sizes are small.

- Distance correlation and Distance Covariance

The distance correlation [14,15] is defined with a random sample $(A,B)=(A_{k},B_{k}):k=1,...,n$, where n is independent and identically distributed for each random pair (*A*,*B*), let the random distribution for *A* be *R*^*p*^ and the random distribution for *B* be *R*^*q*^, the Euclidean distance matrix is calculated with $\left(x_{ij}=|A_{i}-A_{j}{|}_{p}\right)$ and $\left(y_{ij}=|B_{i}-B_{j}{|}_{q}\right)$. with *i*, j=1,..,n. Determine the distance between the elements in the matrix as follows:

$$X_{ij}=x_{ij}-{\overset{―}{x}}_{i.}-{\overset{―}{x}}_{.j}+{\overset{―}{x}}_{...}$$ (1)

where ${\overset{―}{x}}_{i.}=\frac{1}{n}\sum_{j=1}^{n}x_{ij},{\overset{―}{x}}_{.j}=\frac{1}{n}\sum_{i=1}^{n}x_{ij},\overset{―}{x}=\frac{1}{n^{2}}\sum_{ij=1}^{n}x_{ij}$

Similarly, calculating *Y*_*ij*_

$$Y_{ij}=y_{ij}-{\overset{―}{y}}_{i.}-{\overset{―}{y}}_{.j}+{\overset{―}{y}}_{...}$$ (2)

with ${\overset{―}{y}}_{i.}=\frac{1}{n}\sum_{j=1}^{n}y_{ij},{\overset{―}{y}}_{.j}=\frac{1}{n}\sum_{i=1}^{n}y_{ij},\overset{―}{y}=\frac{1}{n^{2}}\sum_{ij=1}^{n}y_{ij}$

Distance Covariance-DisV [15,19]: Distance Covariance of $DisV_{n}(A,B)$ is calculated as follows:

$${DisV}_{n}^{2}(A,B)=\frac{1}{n^{2}}\sum_{i,j=1}^{n}X_{ij}Y_{ij}$$ (3)

Distance Correlation-DisC [15,19]: Distance Correlation *DisC*_*n*_(*A*,*B*) is calculated as follows:

$${DisC}_{n}^{2}(A,B)=\{\begin{array}{cc} \frac{{DisV}_{n}^{2}(A,B)}{\sqrt{{DisV}_{n}^{2}(A){DisV}_{n}^{2}(B)}} & {DisV}_{n}^{2}(A){DisV}_{n}^{2}(B)>0\\ 0, & {DisV}_{n}^{2}(A){DisV}_{n}^{2}(B)=0 \end{array}\}$$ (4)

In which, $DisV_{n}^{2}(A)$ is determined as follows:

$${DisV}_{n}^{2}(A)={DisV}_{n}^{2}(A,A)=\frac{1}{n^{2}}\sum_{i,j=1}^{n}X_{n}^{2}$$ (5)

The values of $DisV_{n}^{2}$ and $DisC_{n}^{2}$ are always non-negative.

- The Bias-Corrected Distance Correlation

The unbiased (squared) Distance Covariance (DCOVU) [15,20] is an estimator of the squared distance covariance. It is based on the inner product definition of distance covariance in the Hilbert space of U-centered distance matrices. The sample sizes (number of rows) of the two samples must match, and the samples should not contain missing values.

The Bias-Corrected Distance Correlation (BCDCOR) [15,20] provides an improved estimate of the squared distance correlation. It corrects for the bias present in the original distance correlation. Similar to dcovU, it does not require taking the square root. Use bcdcor when assessing the association between two variables while accounting for the underlying structure of the data.

With U-centered is the corresponding squared distance covariance statistic, it is an unbiased estimator of the population coefficient. Where *X* = *x*_*ij*_ be a symmetric matrix (n × n). The U-centered matrix $\tilde{X}$ The (*i*,*j*)–th entry of $\tilde{X}$ is:

$${\tilde{X}}_{ij}=\left\{ \begin{array}{cc} x_{ij}-\frac{1}{n-2}\sum_{i=1}^{n}x_{ij}-\frac{1}{\left(n-2\right)}\sum_{j=1}^{n}x_{ij}+\frac{1}{\left(n-1)(n-2\right)}\sum_{i,j=1}^{n}x_{ij} & i\neq j\\ 0, & i=j \end{array}\right.$$ (6)

To calculate the unbiased statistic, the double centering operation is replaced with U-centering, the U-centered distance matrices $\tilde{X}$ and $\tilde{Y}$ is presented in follow:

$$(\tilde{X}*\tilde{Y})=\frac{1}{n(n-3)}\sum_{i\neq j}{\tilde{X}}_{i,j}*{\tilde{Y}}_{i,j}$$ (7)

Bias-corrected distance correlation $DisC_{n}^{2}$ is defined by normalizing the inner product statistic with the bias corrected *DisV* statistics.

## Modeling

### Partitioning data

Cross-validation [21], a crucial technique in machine learning to assess model performance. This approach called rotation estimation or out-of-sample testing, is a model validation technique. It assesses how well the results of a statistical analysis will generalize to an independent data set. Cross-validation is commonly used in prediction tasks to estimate how accurately a predictive model will perform in practice.

The goal of this approach is to test the model’s ability to predict new data that was not used during training. In a prediction problem, the dataset will be split into two parts: Training dataset: Known data used for model training. Validation dataset (testing set): Unknown data against which the model is tested. Cross-validation involves: Partitioning the data into complementary subsets (e.g., training set and validation set). Running the analysis on one subset (training set) and validating it on the other (validation set). Repeating this process with different partitions to reduce variability. Combining (averaging) the results over multiple rounds to estimate the predictive performance of the model. Types of Cross-Validation include: k-fold Cross-Validation: Divides the data into k equally sized folds. The model trains on k-1 folds and validates on the remaining fold, repeating k times. Leave-One-Out Cross-Validation (LOOCV): Each data point serves as a validation set, and the rest are used for training. Stratified Cross-Validation: Ensures class distribution balance across folds. Holdout Method: Splits data into training and validation sets (usually 80-20 or 70-30 split). Nested Cross-Validation: Used for hyper-parameter tuning within each fold.

### Evaluation

Precision and recall [21], two important performance metrics in pattern recognition, information retrieval, and machine learning. Increasing precision often leads to a decrease in recall, and vice versa. Finding the right balance depends on the specific problem and application.

Precision (also known as positive predictive value) measures the fraction of relevant instances among the retrieved instances. Precision is calculated using the following formula:

$$Precision=\frac{TruePositives}{TruePositives+FalsePositives}$$ (8)

True Positives (TP) are the instances correctly identified as belonging to the positive class.

False Positives (FP) are instances incorrectly labeled as positive when they actually belong to the negative class.

Precision reflects the quality of an algorithm’s results. A higher precision means that the algorithm returns more relevant results than irrelevant ones.

Recall (also known as sensitivity) measures the fraction of relevant instances that were retrieved. The formula for recall is

$$Recall=\frac{TruePositives}{TruePositives+FalseNegatives}$$ (9)

True Negatives (TN) are instances correctly identified as irrelevant. False Negatives (FN) are relevant instances that were missed.

Recall reflects the number of relevant results returned by an algorithm. High recall means that most relevant results are captured, regardless of whether some irrelevant ones are also included.

### Recommendation model with distance correlation

**Fig 2:**
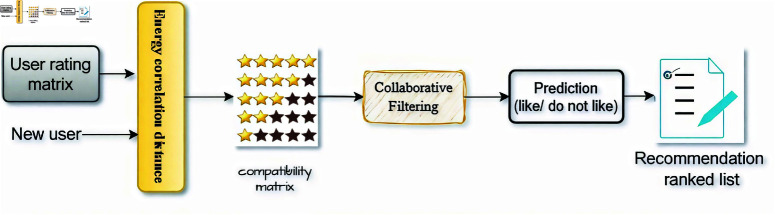
Modeling of collaborative filtering with bias-corrected distance correlation statistic.

Energy–based Collaborative Filtering is a newly adopted method to predict user preferences based on the relationships between the users and between the items. In recent years, recommendation systems have been widely used for applications with increasing amounts of data. Integrating the energy distance into the recommendation system aims to improve the appropriate balance between compatibility and recommendation for the CF models. This model provides personalized recommendations to users and this proposed model has proven the feasibility in the experiment result. Fig 2 clearly presents the working model of the energy-based CF model that includes: 1. The input is the data rating matrix (R×U×I), and the active user vector *u*_*a*_) (*r*_*i*_1, *r*_*i*_2, *r*_*i*_3,.., *r*_*i*_*n*); 2. use the energy approach to calculate compatibility between the objects; 3. In collaborative filtering, the model will predict the missing rating items of the user *u*_*a*_ by formula ${\hat{r}}_{aj}$.; and the output is a list of ratings to recommend.

One popular approach in recommendation systems is collaborative filtering. It leverages user interactions (such as the ratings, the clicks, or the purchases) to make personalized item recommendations. Within collaborative filtering, there are different techniques, including user-based and item-based methods.

### Algorithm user-based recommendation model with bias-corrected distance correlation

Collaborative filtering aims to predict missing elements (e.g., user-item ratings) based on the preferences of similar users. One common formulation is matrix completion, where the recommendation predicts the missing entries in a user-item interaction matrix. The algorithm be expressed with five steps as follows:

**Algorithm 1. UBCF model with bias-corrected distance correlation statistic.**



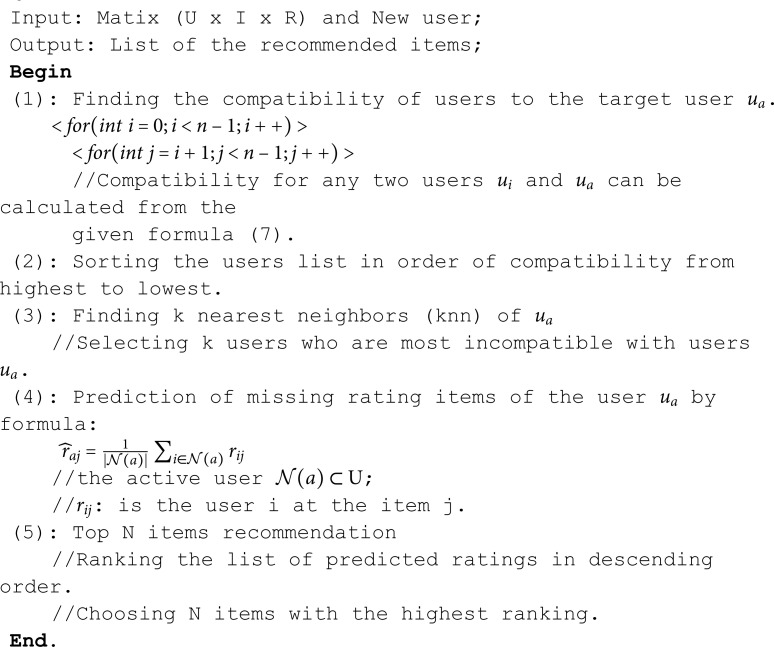



### Algorithm item-based recommendation model with bias-corrected distance correlation

Item-based recommendation model, instead of directly modeling user preferences as UBCF, these models focus on item-item compatibility. The idea of this model with Bias-Corrected Distance Correlation is to recommend items compatible to those a user has already interacted with (e.g., rated, purchased, or viewed). The core assumption is that users who liked similar items in the past will continue to like similar items in the future. Distance correlation is used to measure of dependence between two random variables that captures both linear and nonlinear relationships. The algorithm of the proposed model including three steps as follows:

**Algorithm 2. IBCF model with bias-corrected distance correlation statistic.**



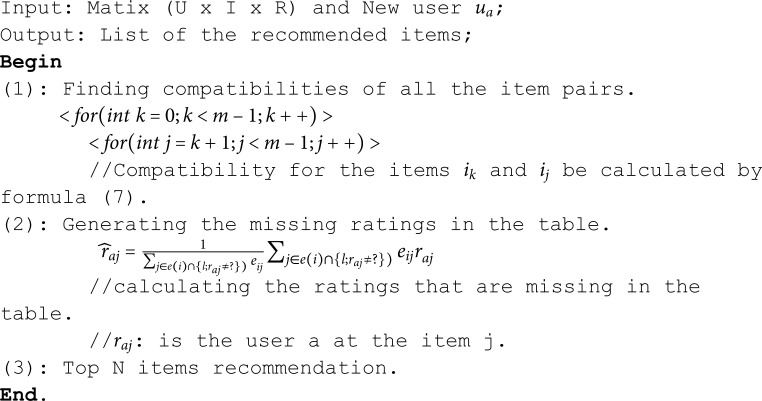



## Experiment

**Fig 3:**
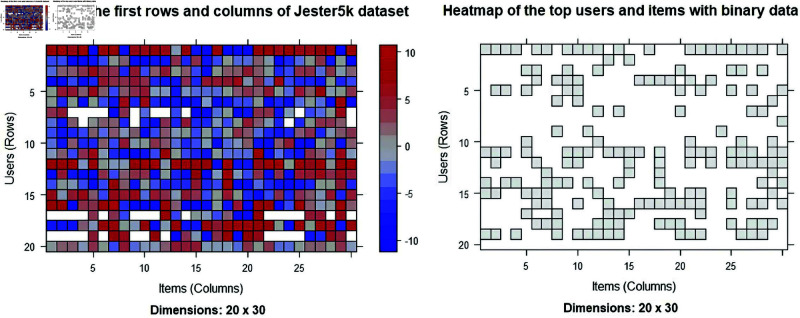
Heatmap of the first 20 users and 30 items of dataset.

In this section, the authors have used functions in R language to present some experiments and to check the accuracy of the proposed model. The packages integrated and used in the article, that are: recommenderlab-1.0.6 (https://cran.r-project.org/web/packages/recommenderlab/index.html), energy-1.7-12 (https://cran.r-project.org/web/packages/energy/index.html), irlba-2.3.3 (https://cran.r-project.org/web/packages/irlba/index.html), proxy-0.4-24 (https://cran.r-project.org/web/packages/proxy/index.html), registry-0.5-1 (https://cran.r-project.org/web/packages/registry/index.html), arules-1.6-6 (https://cran.r-project.org/web/packages/arules/index.html) tool. Two experiments based on datasets commonly used in recommendation systems are: Jester5k (https://rdrr.io/cran/recommenderlab/man/Jester5k.html). A brief description of the dataset is presented as follows:

The Jester5k dataset contains a sample of 5000 users from the anonymous ratings data from the Jester Online Joke Recommendation System collected between April 1999 and May 2003. The format of Jester5k is: Formal class realRatingMatrix [package recommenderlab]. Fig 3 presents the first heatmap of 20 users and 30 items.

**Fig 4:**
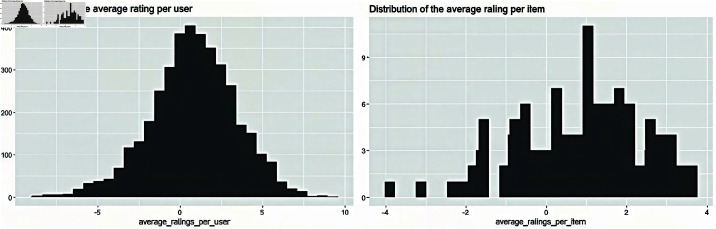
Distributions of the average rating per users and items.

The format of JesterJokes is: vector of character strings. Jester5k contains a 5000 x 100 rating matrix (5000 users (row) and 100 jokes (col)) with ratings value from -10.00 to +10.00. All selected users have rated 36 or more jokes. The data also contains the actual jokes in JesterJokes. Fig 4 depicts the distributions of the average rating per users and items.

### Scenario 1: Bias-corrected-based recommendation with users

In this experimental result, the authors used the Jester5k dataset. Additionally, R language is also used to build the proposed model (UBCF-Bias-distcov) and the compared models (UBCF-Matching, UBCF-Cosine, UBCF-Pearson, UBCF-Phi, UBCF-Dice). The packages are used to build the models for the experiments include: Energy, arules, proxy, recommnederlab, registry, irlba package. The models are tested based on the rating values of the users.

**Fig 5:**
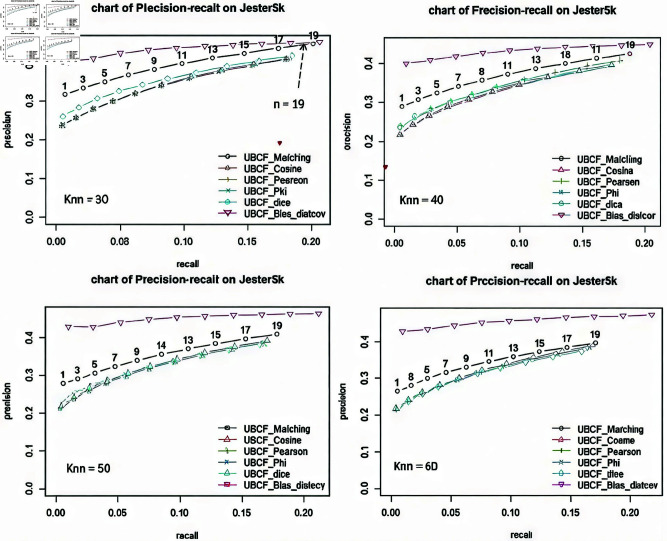
Evaluating the precision-recall for the Bias-Corr-CF with knn = 30, 40, 50, 60.

The results in Fig 5 show that as *n* (number of items to recommend) from 1 to 19, and *knn = 30, 40, 50, 60*. The accuracy of the proposed UBCF-Bias-distcov model is higher than the compared models (UBCF-Matching, UBCF-Cosine, UBCF-Pearson, UBCF-Phi, UBCF-Dice). Specifically, the Precision value of the Bias-Corr-CF model is from 0.4 or higher, while the Precision values of the other models are always lower than 0.4. In addition, the Recall value of the Bias-Corr-CF model is always greater than 0.20, while the compared models are less than 0.20. However, when *knn = 30*, the number of items to recommend *n = 19*, the proposed model’s the accuracy of recommendation ability will not be higher than the accuracy of the UBCF-Matching model.

**Fig 6:**
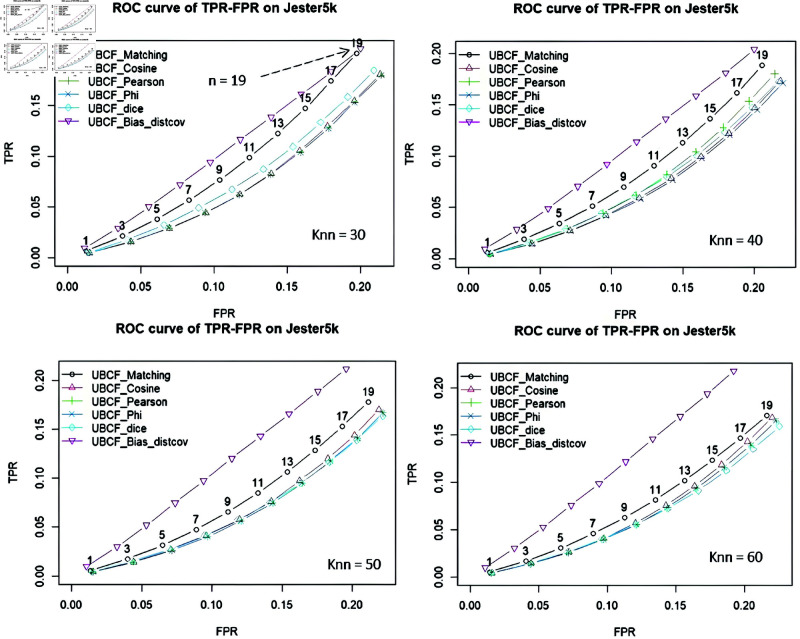
The ROC curve for the Bias-Corr-CF with knn = 30, 40, 50, 60.

Fig 6 shows a plot illustrating the performance of the UBCF-Bias-distcov model, i.e. the ROC (Receiver Operating Characteristics) curve. The results show that with two continuously increasing values of TPR (True Positive Rate) and FPR (False Positive Rate), the area under the curve (ROC) of the proposed model is larger than the other models compared with *knn = 30, 40, 50 , 60*. However, at knn = 30, with the number of items to be recommended (n = 19), the TPR values and the FPR values of the Bias-Corr-CF model are almost equal to the TPR values and the FPR values of the UBCF-Matching model.

### Scenario 2: Bias-corrected-based recommendation with item

In scenario 2, an experiment is conducted to evaluate the classification performance using different evaluation methods. The authors used the Jester5k dataset to evaluate the items-based collaborative filtering recommendation model, which mainly focuses on using the bias-corrected distance correlation Statistic approach. From the confusion matrices, we can calculate the Precision and Recall values. These two common values are used to evaluate the proposed model (IBCF-Bias-distcov) with the compared models (IBCF-Pearson, UBCF-Affinity, IBCF-Karypis, UBCF-Conditional).

**Fig 7:**
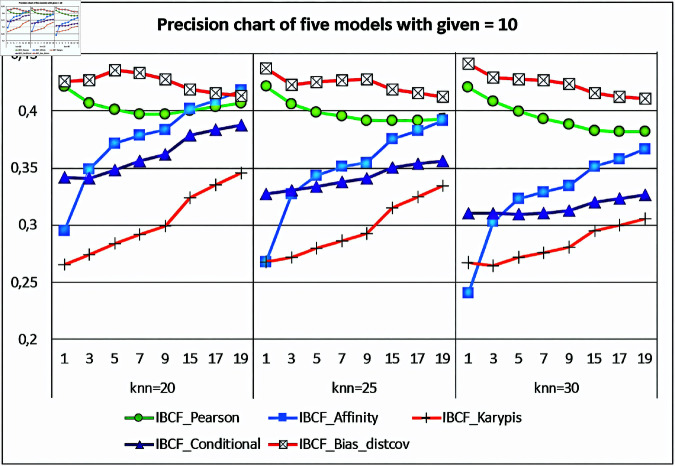
Evaluating the precision values for the five recommendation models with given = 10 (fixed).

As shown, Fig 7 has five curves, one for each model, the first class model (the IBCF-Bias-distcov generated model) obtains a better Precision value than the other four models, when *knn = 20, 25, 30*, and *n = 1,..19*. From the confusion matrices, the authors can calculate the Precision value when each given rating is *given = 10* (fixed) and the k nearest neighbors *knn = 20, 25, 30* (changed).

**Fig 8:**
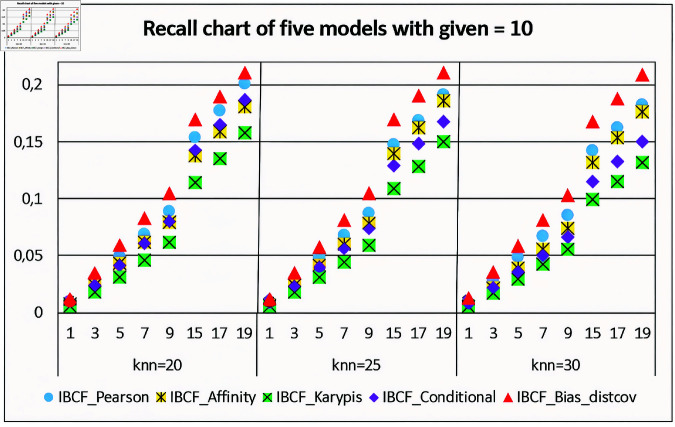
Evaluating the recall values for the five recommendation models given = 10 (fixed).

Fig 8 shows the Recall chart of the five models. With K-nearest neighborhood *k = 50, 70, 100*, and *given = 10* (fixed). The chart shows that when the number of items to be recommended *n = 1, 3, 5, 7, 9, 17, 19* respectively. The Recall values of the proposed model is always higher than the Recall values of the remaining models. However, at *n = 1*, the Recall values of the IBCF-Bias-distcov are not significantly high.

**Fig 9:**
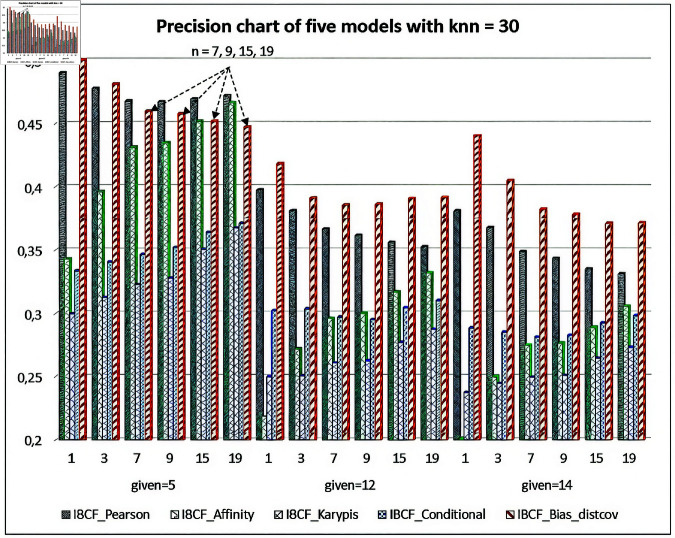
Evaluating the precision values for the five item-based recommendation models.

**Fig 10:**
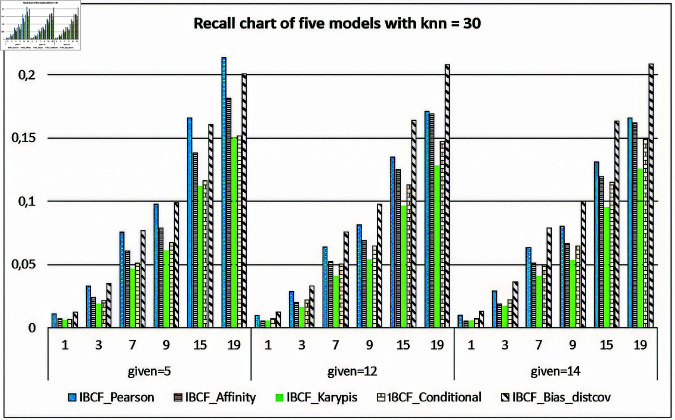
Evaluating the recall values for the five item-based recommendation models.

Figs 9 and 10 present the evaluation of the Precision-Recall values for the five models, when *knn* = 30 (fixed) and given=5,12,14, respectively. The results in the two figures show very clearly, with given=12,14, and the number of the items to recommend n=1,3,7,9,15,19, the Precision values and Recall values of the IBCF-Bias-distcov model are always higher than the four compared models. However, at given=5, the number of items to recommend n=7,9,15,19, both Precision values and Recall values of the proposed model give the low results.

## Discussion

The Bias-Corr-CF model demonstrates superior effectiveness in recommendation tasks through its innovative incorporation of bias-corrected distance correlation statistics. The model’s key strength lies in its ability to capture both linear and non-linear relationships between users and items, addressing a fundamental limitation of traditional correlation-based approaches. By leveraging the bias-corrected distance correlation, the model more accurately measures the true strength of associations in the rating patterns, leading to more reliable similarity computations and subsequently better recommendations. This is particularly evident in the improved precision and recall metrics across different experimental scenarios. While the model faces certain limitations in terms of computational complexity and data requirements, these challenges are outweighed by its ability to generate more accurate recommendations through better statistical modeling of user-item relationships. The integration potential with deep learning, machine learning, and neural graph-based approaches suggests promising directions for enhancing the model’s capabilities while maintaining its core statistical advantages. This combination could lead to more sophisticated recommendation systems that better capture the complex dynamics of user preferences while ensuring statistical robustness and reliability in the recommendation process.

## Conclusion

This paper presents a new recommendation method with a bias-corrected distance correlation approach. Initially, this method creates a compatibility matrix for users or news items in the system based on the ratings. Then, a collaborative filtering method is used to predict the missing ratings. In the experimental part, two scenarios were created to test the results of researching about the compatibility between the users and the compatibility between the items. The proposed model was compared with other recommendation models (the compared models are available in the recommenderlab). Experimental results show that using distance correlation in the recommendation system has improved the quality of the generated recommendations. These results were confirmed through the scenarios 1 and the scenarios 2. The results clearly show that the using of index n (number of news items to suggest) is chosen from 1 to 19 for Bias-corrected-based recommendation with users, along with the neighborhoods this are found by using the kNN algorithm (knn = 30, 40, 50, 60). Besides, for each known rating given = 10 (fixed), knn = 20, 25, 30 (changed), knn = 30 (fixed), given = 5, 12, 14 (changed) for Bias-corrected-based recommendation with items. All have significantly increased the quality of the recommendation generated. However, with the limitation of the proposed model, such as the input data, the methods, and the model execution time is long. In the future, the authors will continue to study and to test the Bias-Corr-CF model on the other datasets, and the other recommendation systems.

## References

[1] Adomavicius G, Tuzhilin A. Toward the next generation of recommender systems: a survey of the state-of-the-art and possible extensions. IEEE Trans Knowl Data Eng. 2005;17(6):734–49. doi: 10.1109/tkde.2005.99

[2] Suresh K, Gorakala. Building a Recommendation System with R [book] Packt Publishing, ISBN: 9781783554492; 2015.

[3] Wang X, He X, Wang M, Feng F, Chua T-S. Neural graph collaborative filtering. In: Proceedings of the 42nd International ACM SIGIR Conference on Uesearch and Uevelopment in Unformation Uetrieval, 2019. 165–74. doi: 10.1145/3331184.3331267

[4] He X, Liao L, Zhang H, Nie L, Hu X, Chua T-S. Neural Collaborative Filtering. In: Proceedings of the 26th International Conference on World Wide Web, 2017. 173–82. doi: 10.1145/3038912.3052569

[5] Zhang S, Yao L, Sun A, Tay Y. Deep Learning Based Recommender System. ACM Comput Surv. 2019;52(1):1–38. doi: 10.1145/3285029

[6] He X, Chen T, Wang Z, Chua TS. Neural Collaborative Filtering with Self-supervised learning. In: Proceedings of the 44th International ACM SIGIR Conference on Research and Development in Information Retrieval, 2021. 1828–32.

[7] Zhao Q, Chen Y, Liu J. Enhancing collaborative filtering with user-item subgroups. IEEE Transactions on Knowledge and Data Engineering. 2023;35(5):4812–25.

[8] Adomavicius G, Manouselis N, Kwon Y. Multi-criteria recommender systems. Recommender Systems Handbook. 2011. p. 769–803.

[9] Schafer BJ, Frankowski D, Herlocker J, Sen S. Collaborative filtering recommender systems. The adaptive web. Berlin, Heidelberg: Springer-Verlag. 2007. p. 291–324.

[10] Schafer JB, Frankowski D, Herlocker J, Sen S. Collaborative Filtering Recommender Systems. In: Brusilovsky P, Kobsa A, Nejdl W, editors. The Adaptive Web. Berlin, Heidelberg: Springer. 2007. p. 291–324.

[11] Bal-chanowski M, Boryczka U. Collaborative rank aggregation in recommendation systems. Procedia Computer Science. 2022;207:2213–22.

[12] Lipovetsky S, Szekely GJ, Rizzo ML, Raton B. The energy of data and distance correlation. Technometrics. 2023;65(3):446–8.

[13] Rizzo ML, Szekely GJ. The distance correlation t-test of independence in high dimension. Journal of Multivariate Analysis. 2013;117:193–213.

[14] Szekely GJ, Rizzo ML, Bakirov NK. Measuring and testing dependence by correlation of distances. Annals of Statistics. 2007;35(6):2769–94.

[15] Kosorok MR. On Brownian Distance Covariance and High Dimensional Data. Ann Appl Stat. 2009;3(4):1266–9. doi: 10.1214/09-AOAS312 20574547 PMC2889501

[16] Park T, Shao X, Yao S. Partial martingale difference correlation. Electron J Statist. 2015;9(1). doi: 10.1214/15-ejs1047

[17] Tran TCT, Phan LP, Huynh HX. Energy-based collaborative filtering recommendation. IJACSA. 2022;13(7). doi: 10.14569/ijacsa.2022.0130766

[18] Tran TCT, Phan LP, Huynh HX. Approach of Item-Based Collaborative Filtering Recommendation Using Energy Distance. JAIT. 2024;15(1):10–6. doi: 10.12720/jait.15.1.10-16

[19] Gao L, Fan Y, Lv J, Shao Q-M. Asymptotic distributions of high-dimensional distance correlation inference. Ann Stat. 2021;49(4):1999–2020. doi: 10.1214/20-aos2024 34621096 PMC8491772

[20] Shen C, Panda S, Vogelstein JT. The Chi-Square Test of Distance Correlation. J Comput Graph Stat. 2022;31(1):254–62. doi: 10.1080/10618600.2021.1938585 35707063 PMC9191842

[21] Herlocker JL, Konstan JA, Terveen LG, Riedl JT. Evaluating collaborative filtering recommender systems. ACM Transactions on Information Systems. 2004;22(1):5–53.

